# Current status and problems of orthopaedic residents in Thailand and Myanmar

**DOI:** 10.12688/mep.18989.1

**Published:** 2022-02-22

**Authors:** Yuichi Kasai, Permsak Paholpak, Taweechok Wisanuyotin, Winai Sirichativapee, Shwe Kyaw Oo, Si Thu

**Affiliations:** 1 Department of Orthopaedics, Khon Kaen University Faculty of Medicine, Khon Kaen, Khon Kaen, 40002, Thailand; 2 Department of Orthopaedic Surgery, University of Medicine 1, Yangon, Yangon, Yangon, Myanmar; 3 Department of Orthopaedic Surgery, Aoyama General Hospital, Toyokawa, Japan

**Keywords:** orthopaedic specialists, orthopaedic residents, medical education, Thailand, Myanmar

## Abstract

**Background:** There is no research about current experiences of orthopaedic residents in Thailand and Myanmar.

**Methods:** A questionnaire survey was distributed among Thai and Myanmar orthopaedic residents to assess their current experiences. This study included a total of 168 participants, comprising 92 (94.8%) of 97 orthopaedic residents in Thailand, and 76 (97.4%) of 78 in Myanmar who answered the questionnaire. The survey comprised nine questions about issues such as the contents of residency training programs, current training satisfaction, and future careers. The survey was administered anonymously between October 2020 and January 2021.

**Results:** Regarding training content, 24 residents reported being "very satisfied", 103 were "satisfied", 37 were "moderately satisfied", and four were "dissatisfied", and respondents spent a mean of 3.1 h/day reading textbooks and research papers. As for salary, five residents answered "satisfied", 46 responded "moderately satisfied", and 117 were "dissatisfied".

**Conclusions:** Many orthopedic residents in Thailand and Myanmar were enthusiastic about and satisfied with their training. Their only problem was that the salary was low.

## Introduction

In Southeast Asian countries, most reports on medical residents becoming specialists have been reported from Singapore ^1– 3^, very few from Thailand ^4^, and none from Myanmar. Moreover, there have been no reports on orthopaedic residents in Thailand or Myanmar in the past.

According to the information from the Royal College of Orthopedic Surgeons of Thailand and Myanmar Orthopaedic Society (Accessed on 4th Mar 2021), the number of orthopaedic specialists was 2,630 in Thailand and 600 in Myanmar, and the ratio of orthopaedic surgeons per 100,000 population is 4.0 in Thailand and 1.2 in Myanmar. As a reference, the number of orthopedic specialists in Japan is 19,598, which amounts to 16.3 per 100,000 population, according to the information from the Japanese Orthopaedic Association website (accessed on 20th Feb 2021). These low numbers of orthopaedic specialists in Thailand and Myanmar may be because the total number of orthopaedic residents in 2021 was limited to 138 in Thailand and 100 in Myanmar (the number of applicants changes between years.)

In the present study, we report a questionnaire survey on Thai and Myanmar orthopaedic residents investigating the actual education conditions in countries with a shortage of doctors and related problems.

## Methods

This study included 168 orthopaedic residents, comprising 92 (18.0 % of all 520 Thai residents) of 97 orthopaedic residents from three universities and three hospitals in Thailand who answered the questionnaire and 76 (28.1% of all 271 residents in Myanmar) of 78 orthopaedic residents from one university in Myanmar who answered the questionnaire. The questionnaire was conducted at the facility of our choice to the orthopedic residents who were attending the lecture on the day of distribution of the questionnaire. In Thailand, 92 out of 97 (94.8%) residents, and in Myanmar, 76 out of 78 residents (97.4%) attended the lecture. The questionnaire survey was created by the first author (YK), and the questionnaire was distributed at the end of the lecture without contacting the participants in advance to conduct this questionnaire. First, the doctor at the facility exposed the purpose of this questionnaire and explained that there would be no advantage or disadvantage to answering this questionnaire, and that this data would be collected, used, and published in an international journal. Then, after answering “yes” to the question asking whether participants consented to answer, all participants answered, and the questionnaire was collected. Windows 10 was used as the software tool, and the data was combined in Excel. The creator of this questionnaire did not collect data.

The mean age of Thai respondents was 28.0 years (ranging 25–31 years), with 77 men and 15 women. Nine residents were married. In contrast, Myanmar orthopaedic residents (Master of Medical Science in Orthopaedics) in this study had a mean age of 30.1 years (26–35 years), with 72 men and four women, and 26 married residents. We conducted two types of questionnaire surveys on these orthopedic residents. One is a questionnaire about the current training, and the other is a questionnaire about hopes for overseas training and expectations for the Japanese Society of Orthopedic Surgery. The results of the latter questionnaire have already been reported ^5^, and thus, this paper describes the results of the former questionnaire survey.

The questionnaire was administered anonymously between October 2020 and January 2021. The following nine questions were asked:

1) Why did you want to be an orthopaedic surgeon?

2) Was the selection test to be an orthopaedic resident difficult? (responses: "very difficult", "difficult", "moderately difficult", or "easy")

3) How long do you sleep per day?

4) How many hours per day do you read orthopaedic textbooks and research papers?

5) What is the most time-consuming task in the training program?

6) Are you satisfied with the training program? (responses: "very satisfied", "satisfied", "moderately satisfied", or "dissatisfied")

7) Are you satisfied with your salary? (responses: "very satisfied", "satisfied", "moderately satisfied", or "dissatisfied")

8) Do you think the orthopaedic specialist examination that you are going to take is difficult? (responses: "very difficult", "difficult", "moderately difficult", or "easy")

9) What is your career plan (or what do you want to do) after passing the orthopaedic specialist examination?

This study adhered to the ethical guidelines and regulations for research on human subjects of Khon Kaen University and was approved by the Ethics committee for human research (Approval number HE641170).　Before answering this questionnaire, its purpose was explained to all participants, as well as the fact that there would be no benefit or disadvantage to providing answers or not. After the oral explanation of the survey contents, only those who answered “yes” to the consent section at the start of the questionnaire were asked to answer; in the end, all residents agreed to participate

## Results

Table 1 shows the results of the questionnaire survey answered by Thai and Myanmar orthopaedic residents.

**Table 1.** Results of the questionnaire survey answered by Thai and Myanmar 168 orthopaedic residents.

Questions	Thailand orthopaedic residents (92 persons)	Myanmar orthopaedic residents (76 persoms)
1. Grounds for being an orthopaedic surgeon	Interest 45 important field of medical care 34 easy to earn money 9 on the advice of seniors 4	interest 43 important field of medical care 25 challenge of new things 5 on the advice of seniors 2 easy to earn money 1
2. Selection test to be an orthopaedic surgery resident	very difficult 14 difficult 53 moderately difficult 23 easy 2	very difficult 7 difficult 60 moderately difficult 9 easy 0
3. Hours of sleep per day	average 5.7 h (4–8 h)	average 6.5 h (5–9 h)
4. Hours of study per day	average 3.5 h (1–11 h)	average 2.6 h (1–5 h)
5. Most time- consuming task in the training program	inpatient treatment plans and postoperative care 49 reading textbooks and research papers 18 surgery 15 physical examinations of outpatients 10	inpatient treatment plans and postoperative care 34 surgery 20 reading textbooks and research papers 12 physical examinations of outpatients 10
6. Satisfaction with the training program	very satisfied 7 satisfied 50 moderately satisfied 32 dissatisfied 3	very satisfied 17 satisfied 53 moderately satisfied 5 dissatisfied 1
7. Satisfaction with the salary	satisfied 4 moderately satisfied 37 dissatisfied 51	satisfied 1 moderately satisfied 9 dissatisfied 66
8. Orthopedic specialist examination	very difficult 24 difficult 52 moderately difficult 16	very difficult 25 difficult 46 moderately difficult 5
9. Future career plan	undecided 61 working at hospital 21 studying a subspecialty 10	undecided 30 studying a subspecialty 20 working at hospital 16 undertaking training abroad 10

The question regarding why they became an orthopaedic surgeon was answered by "interest" by 88 residents, "an important field of medical care" by 59, "easy to earn money" by 10, "on the advice of seniors" by six, and "the challenge of new things" by five participants. The selection test for orthopaedic specialists was rated as "very difficult" by 21 residents, "difficult" by 113 residents, "moderately difficult" by 32 residents, and "easy" by two residents. Mean daily sleep was 6.0 h/day (range, 4–9 h/day), while respondents spent a mean of 3.1 h/day reading textbooks and research papers (range, 1–11 h/day). In the training programs, the most time-consuming tasks were reported to be inpatient treatment plans (including preparing presentation materials such as medical history, present illness, physical examination findings, imaging examination findings, blood test results, diagnosis and treatment schedule for preoperative meetings) and postoperative care by 83 residents, surgery by 35 residents, reading textbooks and research papers by 30 residents, and physical examinations of outpatients by 20 residents. Regarding training contents, 24 residents responded being "very satisfied", 103 were "satisfied", 37 were "moderately satisfied", and four were "dissatisfied". As for salary, five residents answered "satisfied", 46 responded "moderately satisfied", and 117 were "dissatisfied". The difficulty of the orthopaedic specialist examination to be taken was graded as "very difficult" by 49 residents, "difficult" by 98, and "moderately difficult" by 21; no respondents thought the examination would be "easy". The most frequent response for a career plan after passing the examination was "undecided" by 91 respondents, followed by "working at a hospital" by 37 respondents, "studying a subspecialty" by 30 respondents, and "undertaking training abroad" by 10 respondents.

## Discussion

Since orthopaedic diseases include many diseases from trauma to degenerative diseases, the number of patients is very large, and training of orthopedic specialists is important and indispensable in any country, training methods vary widely between countries ^6– 9^. In developing countries such as Thailand and Myanmar, trauma patients with injuries such as fractures are frequently seen, so training as a trauma surgeon is crucial ^10, 11^.

In our study results, orthopeadic residents in Thailand and Myanmar generally trained diligently and were relatively satisfied with the training content. It was speculated that the reason for applying for being orthopaedic specialists was that it was an interesting and important field of medical care, and these might be reasons they were highly motivated to learn. Although passing rates for the selection test to be accepted as orthopaedic residents differ between years and facilities **i.e., universities or hospitals,** the success rate for exams to become orthopaedic residents is 30–70% in Thailand and 10–30% in Myanmar, according to the information from Royal College of Orthopaedic Surgeons of Thailand and Myanmar Orthopaedic Society (Accessed on 4th Mar 2021). Because Thai and Myanmar residents are learning after successfully overcoming a competitive environment, they might have pride and a strong sense of mission to work as one of the few orthopaedic surgeons, which apparently encourages them to study hard. As a result of their hard work, around 95% of residents pass specialist examinations in Thailand and Myanmar, according to the information from the Royal College of Orthopaedic Surgeons of Thailand and the Myanmar Orthopaedic Society (Accessed on 4th Mar 2021).

On the other hand, the low salary was raised as a problem. In addition, relatively many people had not decided their future career plan, which can be attributed to about half of them being first or second-year residents. Treating fractures, dislocations and ligament injuries is the main focus of orthopedic surgery for residents in Thailand and Myanmar, because trauma is overwhelmingly more frequent than non-traumatic diseases ^12, 13^; therefore, it was thought that one of the reasons for this was that few people would like to subspecialise in orthopaedic surgery such as joint surgery, spine surgery, and hand surgery.

 The limitations of this paper are that it is the result of surveying about 20% of residents in Thailand and about 30% of residents in Myanmar, and that there were no question items about the number of years of experience of orthopaedic residents. And, one university in Myanmar, three universities in Thailand, and three hospitals chose facilities that facilitate the questionnaire survey by the authors. Universities in the largest cities in Myanmar and universities and hospitals in local cities in Thailand have been selected, and it is possible that this questionnaire survey does not accurately reflect the situation in each country. However, in the present study, we described orthopedic residents' current situation and problems in Thailand and Myanmar. As further research, we would like to increase the number of respondents and consider the differences relating to participants’ years of experience as residents.

## Conclusion

Many orthopedic residents in Thailand and Myanmar were enthusiastic about and satisfied with their training. The only problem identified was that the salary was low.

## Data availability

### Underlying data

Figshare: All DATA.xlsx, https://doi.org/10.6084/m9.figshare.19083407.v1 ^6^

### Extended data

Figshare: question for Orthopaedic residents.docx, https://doi.org/10.6084/m9.figshare.19100036.v2 ^7^

Data are available under the terms of the Creative Commons Zero "No rights reserved" data waiver (CC0 1.0 Public domain dedication).

## References

[1] Fong JMN Tan YTW Sayampanathan AA : Impact of financial background and student debt on postgraduate residency choices of medical students in Singapore. *Singapore Med J.* 2018;59(12):647–651. 10.11622/smedj.2018068 29876578 PMC6301873

[2] Low JM Tan MY See KC : Sleep, activity and fatigue reported by Postgraduate Year 1 residents: a prospective cohort study comparing the effects of night float versus the traditional overnight on-call system. *Singapore Med J.* 2018;59(12):652–655. 10.11622/smedj.2018036 29552687 PMC6301877

[3] Ong AM Fong WW Chan AK : Using the Postgraduate Hospital Educational Environment Measure to Identify Areas for Improvement in a Singaporean Residency Program. *J Grad Med Educ.* 2019;11(4 Suppl):73–78. 10.4300/JGME-D-19-00234 31428261 PMC6697311

[4] Puranitee P Stevens FFCJ Pakakasama S : Exploring burnout and the association with the educational climate in pediatric residents in Thailand. *BMC Med Educ.* 2019;19(1):245. 10.1186/s12909-019-1687-7 31277615 PMC6612205

[5] Kasai Y Paholpak P Wisanuyotin T : Survey findings of orthopaedic residents in Thailand and Myanmar - Suggestions for international roles of the Japanese Orthopaedic Association. *J Orthop Sci.* 2021;26(6):1135–1137. 10.1016/j.jos.2021.07.022 34497009

[6] Kasai Y Paholpak P Wisanuyotin T : All DATA.xlsx. *figshare*. Dataset.2022. 10.6084/m9.figshare.19083407.v1

[7] Kasai Y Paholpak P Wisanuyotin T : question for Orthopaedic residents.docx. *figshare*. *Journal contribution.* 2022. 10.6084/m9.figshare.19100036.v2

[8] LaPorte DM Tornetta P Marsh JL : Challenges to Orthopaedic Resident Education. *J Am Acad Orthop Surg.* 2019;27(12):419–425. 10.5435/JAAOS-D-18-00084 30480589

[9] Fayaz HC Smith RM Ebrahimzadeh MH : Improvement of Orthopedic Residency Programs and Diversity: Dilemmas and Challenges, an International Perspective. *Arch Bone Jt Surg.* 2019;7(4):384–396. 31448318 PMC6686073

[10] Brouillette MA Kaiser SP Konadu P : Orthopedic surgery in the developing world: workforce and operative volumes in Ghana compared to those in the United States. *World J Surg.* 2014;38(4):849–857. 10.1007/s00268-013-2314-0 24218152

[11] Bunogerane GJ Taylor K Lin Y : Using Touch Surgery to Improve Surgical Education in Low- and Middle-Income Settings: A Randomized Control Trial. *J Surg Educ.* 2018;75(1):231–237. 10.1016/j.jsurg.2017.06.016 28712686

[12] Mahaisavariya B : Musculoskeletal trauma service in Thailand. *Clin Orthop Relat Res.* 2008;466(10):2323–2328. 10.1007/s11999-008-0385-2 18629597 PMC2584308

[13] Abioye IA Ibrahim NA Odesanya MO : The future of trauma care in a developing country: interest of medical students and interns in surgery and surgical specialties. *Int J Surg.* 2012;10(4):209–212. 10.1016/j.ijsu.2012.03.003 22449830

