# Supplementary material for: Current status and problems of orthopaedic residents in Thailand and Myanmar
Source: MedEdPublish (2016). 2022 Jun 16;12:10. Originally published 2022 Feb 22. [Version 2] doi: 10.12688/mep.18989.2 (PMC9370081; doi:10.12688/mep.18989.2)
Supplement: Peer review history

# Peer review: Reviewer response for version 1

**DOI:** 10.21956/mep.20346.r31859

**Published:** 2022-04-12

**Authors:** Hiroshi Ozawa

**Affiliations:** 1 Department of Orthopaedic Surgery, Tohoku Medical and Pharmaceutical University, Sendai, Japan

In the present study, a questionnaire survey was performed for Thai and Myanmar orthopaedic residents to assess their perception of resident programs. This study included a total of 168 residents (92 in Thailand, and 76 in Myanmar). The survey included the satisfaction with training programs, salary, future careers, and so on. In the results, 24 were "very satisfied", 103 were "satisfied", and 37 were "moderately satisfied" with their training programs. Respondents spent a mean of 3.1 h/day reading textbooks and research papers. As for salary, 117 residents answered "dissatisfied". The authors concluded that many orthopedic residents in Thailand and Myanmar were enthusiastic and satisfied with their training. Their problem was the low salary.

 This paper is very interesting. The first author is a Japanese orthopaedic surgeon who has been involved in orthopedics in Thailand and Myanmar for many years and is familiar with the region. As the authors state, there is a shortage of orthopaedic surgeons in Thailand and Myanmar. Many citizens are not able to have enough access to orthopaedic care. This paper showed that young physicians were eager to become orthopedic surgeons and are enthusiastic about their orthopaedic resident training. They studied for more than 3 hours daily except for hospital work.

 This paper seems to be in the interests of the readership and is considered worthy of publication in the journal.

1. The reader would be better able to understand the background of Thailand and Myanmar residency systems if they were explained in the paper. For example, how many years of residency training are required in Thailand and Myanmar? How do the residents train? Is there any national training system? Are there any differences between the Thai and Myanmar training systems?
2. As for the demographics of respondents, please describe their resident grade and what type of hospital they belonged to.
3. It would be better to list the % together with the raw number.

Have any limitations of the research been acknowledged?

Yes

Is the study design appropriate and does the work have academic merit?

Yes

Is the work clearly and accurately presented and does it cite the current literature?

Yes

If applicable, is the statistical analysis and its interpretation appropriate?

Yes

Are all the source data underlying the results available to ensure full reproducibility?

Yes

Are the conclusions drawn adequately supported by the results?

Yes

Are sufficient details of methods and analysis provided to allow replication by others?

Yes

Reviewer Expertise:

Spine

I confirm that I have read this submission and believe that I have an appropriate level of expertise to confirm that it is of an acceptable scientific standard.

# Response

**Published:** 2022-04-28

**Authors:** Yuichi Kasai

**Affiliations:** Khon Kaen University, Thailand

Thank you very much for your useful comments and suggestions. The authors have answered your questions, and the additional changes and sentences in our manuscript were shown in bold.

 For reviewer #2:

*The reader would be better able to understand the background of Thailand and Myanmar residency systems if they were explained in the paper. For example, how many years of residency training are required in Thailand and Myanmar? How do the residents train? Is there any national training system? Are there any differences between the Thai and Myanmar training systems?*

- **Four years of training are required in Thailand and Myanmar. Each training system was created by national organizations: the Royal College of Orthopedic Surgeons of Thailand in Thailand, and the Myanmar Orthopaedic Society in Myanmar. Both countries provide education and clinical practice according to the training program shown annually for each year's residents. In Myanmar, the orthopedic training period was changed from 3 years to 4 years in 2018, and there is no big difference between the training systems in Thailand and Myanmar now. The authors added these sentences to the section of the Introduction.**

*As for the demographics of respondents, please describe their resident grade and what type of hospital they belonged to.*

- **In Thailand, 76 residents belong to university hospitals and 16 people belong to public hospitals. There are 31 first-year residents, 28 second-year residents, 24 third-year residents, and 9 fourth-year residents. In Myanmar, all of them belong to university-related hospitals. There are 29 first-year residents, 27 second-year residents, 20 third-year residents, and 0 fourth-year residents. The authors added these sentences to the section on Methods.**

*It would be better to list the % together with the raw number.*

- **Percentages display has been added to all data.**

# Peer review: Reviewer response for version 1

**DOI:** 10.21956/mep.20346.r31818

**Published:** 2022-03-30

**Authors:** Win Myint Oo

**Affiliations:** 1 Faculty of Medicine, SEGi University, Petaling Jaya, Malaysia; 2 Clinical Campus, Hospital Sibu, Sibu, Sarawak, Malaysia

Congratulations to all authors for your great work!

 This cross-sectional study was conducted among orthopaedic residents in Thailand and Myanmar from October 2020 to January 2021. It was intended to explore their current situations, including their perceptions of the training programme, their future career plan and satisfaction with their salary.

 Altogether 168 orthopaedic residents consisting of 92 from Thailand and 76 from Myanmar were recruited for the study. The mean age of Thai respondents was 28.0 years (ranging from 25–31 years), with 77 men and 15 women. Nine residents were married. In contrast, Myanmar orthopaedic residents (Master of Medical Science in Orthopaedics) in this study had a mean age of 30.1 years (26–35 years), with 72 men and 4 women; among Myanmar participants, 26 were married. The majority (164/168 or 97.6%) reported that they were satisfied with their training programme, while almost 70% (117/168 or 69.6%) were dissatisfied with the salary they received. The most frequent response for a career plan after passing the examination was “undecided” by 91 respondents (54.2%), followed by “working at a hospital” by 37 respondents (22.0%), “studying a subspecialty” by 30 respondents (17.9%), and “undertaking training abroad” by 10 respondents (6.0%). Almost all (98.8%) felt that the selection test for the orthopaedic residency (specialist training) programme was difficult. The average duration of sleep and reading textbooks/research papers during their current training were 6 hours/day and 3.1 hours/day, respectively. All reported that the orthopaedic specialist examination going to be taken will be difficult. In the training programs, the most time-consuming tasks were reported to be inpatient treatment plans and postoperative care. Therefore, it could be concluded that many orthopaedic residents in Thailand and Myanmar were enthusiastic about and satisfied with their training. The only problem identified was receiving a low salary.

 This is an interesting, informative and useful study. There are no significant numbers of research concerning the current experiences of orthopaedic residents in South East Asian countries, especially Myanmar and Thailand. This study revealed the current situation, including the perceptions of orthopaedic residents on the training program and their future career plans. It also explored the problem they encountered or dissatisfiers with their job.

 This study could relay some important feedback to health administrators or policymakers concerned in order to improve the current situation for them.

 However, I would like to give the following suggestions:

1. Some findings like the mean ages and gender distribution of the respondents are described in the “Methods” section. It is better to reveal these in the “Results” section.
2. It is better to show the percentages (for each response) in Table 1 except for numbers 3 (hours of sleep per day) and 4 (hours of study per day).
3. You should discuss with your statistician whether you could use inferential statistics such as a Chi-squared test and/or t-test to check if there is/are any significant difference(s) between (residents from) Thailand and Myanmar.
4. For the findings below Table 1, it is better to reveal the percentages in parentheses. For example, regarding training contents, 24 residents (14.3%) responded as being very satisfied, 103 (61.3%), etc.
5. Check whether reference number 4 is used in this study. I don't see any in-text citation of it. Maybe my mistake.

Have any limitations of the research been acknowledged?

Yes

Is the study design appropriate and does the work have academic merit?

Yes

Is the work clearly and accurately presented and does it cite the current literature?

Yes

If applicable, is the statistical analysis and its interpretation appropriate?

Yes

Are all the source data underlying the results available to ensure full reproducibility?

Yes

Are the conclusions drawn adequately supported by the results?

Yes

Are sufficient details of methods and analysis provided to allow replication by others?

Yes

Reviewer Expertise:

Infectious and non-infectious disease control, Epidemiology of CDs and NCDs, Reproductive Health, Maternal and Child Health

I confirm that I have read this submission and believe that I have an appropriate level of expertise to confirm that it is of an acceptable scientific standard.

# Response

**Published:** 2022-04-28

**Authors:** Yuichi Kasai

**Affiliations:** Khon Kaen University, Thailand

Thank you very much for your useful comments and suggestions. The authors have answered your questions, and the additional changes and sentences in our manuscript were shown in bold.

 For reviewer #1:

*Some findings like the mean ages and gender distribution of the respondents are described in the “Methods” section. It is better to reveal these in the “Results” section.*

- **Thank you very much for your comments. The authors changed the order of the sentences in the section ‘Methods’.**

*It is better to show the percentages (for each response) in Table 1 except for numbers 3 (hours of sleep per day) and 4 (hours of study per day).*

- **In Table 1, the percentages were also listed together with the number of residents.**

*You should discuss with your statistician whether you could use inferential statistics such as a Chi-squared test and/or t-test to check if there is/are any significant difference(s) between (residents from) Thailand and Myanmar.*

- **Regarding the data of residents in Thailand and Myanmar, questions 3 and 4 were compared statistically using a t-test, and other questions were done by a Chi-squared test, and p<0.05 were judged to be significantly different. As a result of comparing the data of Thailand and Myanmar, there was no statistically significant difference. So, the authors added some sentences.**

*For the findings below Table 1, it is better to reveal the percentages in parentheses. For example, regarding training contents, 24 residents (14.3%) responded as being very satisfied, 103 (61.3%), etc.*

- **The percentages were also listed together with the number of residents in the section of ‘Results’.**

*Check whether reference number 4 is used in this study. I don't see any in-text citation of it. Maybe my mistake.*

- **Reference 4 is cited in the first sentence of the Introduction: "In Southeast Asian countries, most reports on medical residents becoming specialists have been reported from Singapore ^1–3^, very few from Thailand ^4^, and none from Myanmar".**
